# A practical approach to vitamin and mineral supplementation in food allergic children

**DOI:** 10.1186/s13601-015-0054-y

**Published:** 2015-03-10

**Authors:** Rosan Meyer, Claire De Koker, Robert Dziubak, Ana-Kristina Skrapac, Heather Godwin, Kate Reeve, Adriana Chebar-Lozinsky, Neil Shah

**Affiliations:** Department Gastroenterology, Great Ormond Street Hospital for Children NHS foundation Trust, London, UK; Department Nutrition and Dietetics, Chelsea and Westminster Hospital NHS Foundation Trust, London, UK; Institute of Child Health, University College London, London, UK

**Keywords:** Dietary adequacy, Elimination diet, Food diary, Non-IgE mediated allergy, Vitamin and mineral supplementation

## Abstract

**Background:**

The management of food allergy in children requires elimination of the offending allergens, which significantly contribute to micronutrient intake. Vitamin and mineral supplementation are commonly suggested as part of dietary management. However a targeted supplementation regime requires a complete nutritional assessment, which includes food diaries. Ideally these should be analysed using a computerised program, but are very time consuming. We therefore set out to evaluate current practice of vitamin and mineral supplementation in a cohort of children with non-Immunoglobulin E (IgE) mediated food allergies.

**Methods:**

This prospective, observational study recruited children aged 4 weeks – 16 years, who required to follow an elimination diet for non-IgE mediated allergies. Only children that improved according to a symptom score and were on a vitamin and/or mineral supplement were included. A 3-day food diary including vitamin and mineral supplementation was recorded and analysed using Dietplan computer program. We assessed dietary adequacy with/without the supplement using the Dietary Reference Values.

**Results:**

One hundred-and-ten children had completed food diaries and of these 29% (32/110) were taking vitamin and/or mineral supplements. Children on hypoallergenic formulas were significantly (p = 0.007) less likely to be on supplements than those on alternative over-the-counter milks. Seventy-one percent had prescribable supplements, suggested by a dietitian/physician. Sixty percent of those without a vitamin supplement had a low vitamin D intake, but low zinc, calcium and selenium was also common. Of the supplemented cohort many continued to be either under or over-supplemented.

**Conclusion:**

This study has raised the question for the first time, whether clinicians dealing with paediatric food allergies should consider routine vitamin and/or mineral supplements in the light of deficient intake being so common in addition to being so difficult to predict.

## Introduction

Fundamental to the management of food allergy in early childhood is the total elimination of offending allergens [1]. These often include cow’s milk, soya, hen’s egg, wheat, fish and nuts; foods which contribute significantly to dietary vitamin and mineral intake [2,3]. Low micronutrient intakes as a result of a dietary elimination have been reported in food allergic children; increasing both the risk of vitamin and mineral deficiency and associated functional morbidity [4-7]. Vitamin D, calcium and omega-3 fatty acids are well documented to be deficient micronutrients in children with IgE-mediated food allergies [8], whilst intakes of trace elements including zinc, selenium and magnesium have been found to be of concern in some non-IgE mediated conditions including allergic colitis and atopic dermatitis [7,9].

A nutritional assessment by a qualified dietitian of the allergic child is now recognised by several international guidelines as essential to ensure dietary adequacy and to support parents in finding suitable alternatives [10-12]. The ideal dietetic assessment within the time constraints of a consultation should include a growth assessment, biochemical (when available) and dietary intake assessment to guide individualised dietary advice [13]. A dietary intake assessment can be very time consuming and is therefore often limited to a 24-hour recall. These dietary recall methods provide only a snap-shot of intake and are often biased [14,15], but are used in practice because they are quick and easy to do, and they provide the baseline for dietetic recommendations including supplementation of vitamin and/or minerals.

Although dietary intake as assessed by a 3-day food diary or nutritional status measured by blood markers, provide more accurate reflections of usual intake and nutritional status, these also have limitations [16,17]. Food diaries are time consuming and complex and therefore require highly motivated parents with a high level of literacy and understanding to cooperate [15,16]. The limitations for blood markers include nutrient plasma or serum concentrations not being a reliable reflection of intake and tissue stores, requiring a large blood sample from a small child and measurements can be affected by the measuring methods, contamination and health of the child at measurement [16,17]. In practice, blood investigations are mostly not available or results not accessible for the majority of cases at the time of the dietetic appointment. Therefore empirical vitamin and or mineral supplementation is currently based on the type of dietary elimination and intake as assessed during the consultation. We therefore set out to evaluate current practice of vitamin and mineral supplementation in a cohort of children with non-Immunoglobulin E (IgE) mediated food allergies, to assess the impact of micronutrient supplementation on vitamin and mineral intake and also whether those with low intakes based on food intake diaries received supplementation.

## Methods

### Subjects and study design

A prospective, observational study was performed in the gastroenterology department, at Great Ormond Street Hospital for Children NHS Foundation Trust, London, UK. Ethical approval (number 11/LO/1177) was obtained for this study. Parents of children aged 4 weeks – 16 years, who had no concomitant non-atopic co-morbidities (i.e. Cerebral Palsy), and required to follow an elimination diet for the diagnosis of suspected food protein induced gastrointestinal allergies, were approached to take part in the study. These children were only included if symptoms improved following the elimination diet as judged by a Likert Scale gastro-intestinal symptom questionnaire, that has previously been developed by the same research team [18]. This questionnaire was administered at baseline prior to commencing the elimination diet and again at 4 weeks after commencing the dietary elimination. In addition to this criterion, the cohort we report on was also on a vitamin and/or mineral supplement.

### Dietary intake

A 3-day estimated food diary (1 weekend day and 2 week days) was recorded, at a minimum of 4 weeks following the initiation of the exclusion diet. Carers were given detailed instructions on how to complete the diary as accurately as possible, including a portion size guide and a sample menu. Hypoallergenic formula (HF) consumption (including type and volume), milk alternatives (i.e. oat, rice or almond milk) for older children and vitamin and mineral supplements (suggested by dietitian/physician or self-initiated) were also documented. Both over-the-counter and prescribed supplements were further categorised as: calcium, calcium and vitamin D, multivitamin, multivitamin and mineral, iron, and a combination of the aforementioned. All infants that were exclusively breastfed or received ≥ 2 breast feeds per day in addition to their HF were excluded from the dietary analysis, due to difficulties in estimating breast milk consumption in the individual patient. We choose this method, due to studies by Lanigan et al. [19] indicating that a weighed record does not give a significant advantage to an estimated record and also following the study by Ocke et al. [20] indicating a 3-day food dairy being a feasible method in European children.

Food diaries were discussed with parents and any unclear entries were clarified by the research dietitian at the time of the study appointment or by telephone communication after the appointment. The UK Food Portion Sizes published by the Food Standard Agency was used to help guide parents and healthcare professionals in estimating the correct portion size whenever portions needed converting from household measures to grams [21].

Nutritional intake data was assessed using Dietplan 6 Software (Forestfield Software Limited, UK). Any foods, in particular specialist foods free from allergens, as well as HF and vitamin and mineral supplements not available on the software database were manually added by the researcher, and product information was obtained from the manufacturer where necessary.

We assessed the impact of the vitamin and mineral supplements on dietary intake by using the UK Dietary Reference Values in the following way: inadequate intake was defined as achieving less than the Lower Reference Nutrient Intake (LRNI – meeting nutrient requirements for 2.5% of population) and adequate intake as achieving the Reference Nutrient Intake (range between LRNI and excessive intake) [22,23]. There is currently no RNI for vitamin D, for children ≥ 4 years of age in the UK; as a result we used the recommendations from the UK Department of Health which recommended 7 – 8.5 mg for children until 5 years of age and for older children we used 10 ug/day as reference value.

As there is paucity of data regarding Safe Upper Limits for many of the micronutrients in children, we arbitrarily defined excessive intake as more than 200% of the RNI [23].

### Biochemical markers of micronutrient intake

We also collected biochemical markers of micronutrient intake, where available, that were performed at Great Ormond Street Hospital for Children, taken within 3 months of the 3 day food diary. As this hospital is a tertiary referral centre, many children may have had biochemical markers of micronutrient intake in their local centres, which we did not have access to.

### Statistical analysis

Statistical analysis was performed using IBM SPSS Statistics for Windows, version 22 (Armonk, NY). Continuous and categorical data are described respectively as medians and interquartile range, and percentages and rates. In order to establish the impact of vitamin and mineral supplementation on micronutrient intake, food diaries of those children receiving a vitamin and mineral supplement were compared to the Dietary Reference Ranges with and without the supplements. The Pearson Chi-Square test was used to compare the differences in proportions of gender between groups receiving and not receiving VMS; children on vitamin and mineral supplements that were on a HF/over-counter-milk. The Fisher exact test was used to compare rates of dietary eliminations. Mann–Whitney *U* test was used to compare age between groups receiving and not receiving VMS; and to compare RNI intake of vitamin A and vitamin D between children taking different supplements. All tests were two-sided and significance level was set at 0.05.

## Results

We recruited 131 children for this study, but 110 children had a complete food diary and of these 29% (32/110) were taking vitamin and/or mineral supplements. The supplemented group included 21 boys (65.6%) and the median age of this group was 5.1 years (IQR: 1.5 to 8.5). Although the proportion of boys was similar in supplemented and un-supplemented group (p 0.916) the age of the supplemented group was significantly higher (p = 0.005) than the median age of the group not receiving any supplementation (1.6, IQR: 0.7 to 4.6) [Table 1]. A total of 9/32 children on supplements (28%) were established on a HF, 17/32 (53%) on over-the-counter milk alternatives (i.e. coconut milk, oat milk or rice milk) and 6 (19%) had no cow’s milk alternative. We found that children on over-the-counter milk alternatives/no milk replacement were significantly more likely to be on supplements compared to those on a HF in the whole cohort (n = 110): 40.4% (23/57) vs. 17.0% (9/53), p = 0.007.The majority of children on supplements (68%, n = 22/32) had prescribable supplements, consisting of mainly multivitamins, calcium and vitamin D and in two cases an iron supplement. The remaining 12 children (2 overlapped between categories) were taking over-the-counter preparations of multivitamins and also omega-3-fatty acids. Table 2 outlines the type of supplements used. In 23/32 (71%) the suggestion for a vitamin and/or mineral supplement was made by the dietitian/physician, in 6 cases both the dietitian and the parents were involved in choosing the supplement and in 3 children the parents started a supplement without dietetic input. From the cohort on supplements, 14 children were eliminating ≥ 3 foods (cow’s milk, soya and gluten/egg/ other), 10 eliminated 2 foods (milk and soya) and 8 excluded 1 food only (i.e. cow’s milk). Tables 1 and 2 describe this cohort in further detail, including the elimination diets and the availability of biochemical markers of micronutrient intake blood markers in this cohort.

**Table 1:** **Demographic details, including dietary elimination of the population with and without vitamin supplementation**

**Description**	**VMS**	**Without VMS**	**p value**
Age	5,1 years	1.6 years	0.005**
Male	21/32 (66%)	52/78 (67%)	0.916
*Dietary Elimination*			
Cow’s Milk	6 (19%)	5 (6%)	0.077
Cow’s milk and soya	9 (28%)	11 (14%)	0.104
Cow’s milk, soya and egg	3 (9%)	5 (6%)	0.689
Cow’s milk, soya, egg and wheat/gluten	5 (16%)	9 (12%)	0.544
Other*	9 (28%)	48 (62%)	0.002**

*This group consists of children with other combinations of food eliminations, **p < 0.01.

**Table 2:** **Dietary supplementation and biochemical markers of micronutrient intake of the group of children receiving supplements**

**Description**	**Patient numbers**
*Type of Supplement* (some may have multiple supplements)	
Calcium	5
Calcium and vitamin D	4
Multivitamin (containing vitamin D)	15
Multivitamin and Mineral	8
Therapeutic iron	2
Other	5
*Supplement initiated by*:	
Dietitian/Physician	23
Dietitian and parent	6
Parent	3
*Biochemical markers of micronutrient intake*: (some children	
had multiple biochemical markers taken)	
Total available	7
Iron profile	7
Selected vitamins	2
Selected trace elements	3
*Deficiencies:*	
Iron	2
Zinc	1

When we assessed dietary adequacy of micronutrients without a supplement (n = 110), based on the definitions outlined in the methodology, we found that no child in the whole group had a deficient vitamin C intake and that low intakes of vitamin B12, B6, thiamine and folate were rare (Table 3). The dietary intake also revealed that in the whole group 60% (66/110) had a low vitamin D intake and that low zinc, copper and selenium were also seen in some patients (Table 3).

**Table 3:** **Overview of the vitamin and mineral supplementation: deficient intake in the whole cohort and those that received supplementation and the impact of the supplementation on dietary adequacy**

**Nutrient**	**Total number of children receiving supplements for each micronutrient**	**Deficient intake without VMS (whole cohort)**	**Deficient patients who were taking VMS** ^**§**^	**>200% RNI with VMS**	Adequate intake* with VMS	**Intakes < LRNI with VMS**
**Vitamin C**	23	0% (0/110)	N/A (0/0)	96% (22/23)	4% (1/23)	0% (0/23)
**Vitamin A**	22	10% (11/110)	9% (1/11)	59% (13/22)	41% (9/22)	0% (0/22)
**Niacin**	21	8% (9/110)	22% (2/9)	62% (13/21)	38% (8/21)	0% (0/21)
**Vitamin B6**	21	2% (2/110)	0% (0/2)	95% (20/21)	5% (1/21)	0% (0/21)
**Thiamin**	18	2% (2/110)	0% (0/2)	89% (16/18)	11% (2/18)	0% (0/18)
**Calcium**	18	9% (10/110)	30% (3/10)	44% (8/18)	50% (9/18)	6% (1/18)
**Vitamin B12**	17	1% (1/110)	100% (1/1)	94% (16/17)	6% (1/17)	0% (0/17)
**Riboflavin**	16	6% (7/110)	14% (1/7)	63% (10/16)	38% (6/16)	0% (0/16)
**Vitamin D**	25	60% (66/110)	27% (18/66)	16% (4/25)	44% (11/25)	40% (10/25)
**Folate**	12	3% (3/110)	0% (0/3)	67% (8/12)	33% (4/12)	0% (0/12)
**Iron**	10	7% (8/110)	0% (0/8)	40% (4/10)	60% (6/10)	0% (0/10)
**Zinc**	7	20% (22/110)	9% (2/22)	14% (1/7)	86% (6/7)	0% (0/7)
**Copper**	5	10% (11/110)	0% (0/11)	20% (1/5)	80% (4/5)	0% (0/5)
**Selenium**	3	11% (12/110)	0% (0/12)	67% (2/3)	33% (1/3)	0% (0/3)

*Children that had > LRNI and < 200% of RNI were classified as having adequate intake.

^§^This is the number of children from the whole cohort that had a deficient intake and were on a VMS.

We then assessed dietary intake in those children with a supplement (n = 32) and whether those who had a low micronutrient intake also received appropriate vitamin and/or mineral supplementation. Our data suggests that although 25 children did receive a supplement with vitamin D, only 18/66 (27%) from the cohort with insufficient intake, were correctly identified as requiring this supplement and 10 of those receiving the supplement continued to not achieve their vitamin D requirements. We also found that 22 children had a low zinc intake however only 2/22 of this cohort was given a supplement containing zinc. Similarly, from the whole cohort 10 were identified as having a low calcium intake according to the food diary, but from this group only 3 children received a calcium supplement. However, 15 (18 in total) other children with an adequate calcium intake, were given a calcium supplement (Table 3), which is a similar trend for other vitamins and minerals.

Many children had an intake with supplementation exceeding 200% of the RNI for vitamin C, vitamin B6, thiamin, folate, selenium and vitamin A. In fact we found that of the 22 patients on a supplement containing vitamin A, 59% (n = 13) had intakes exceeding 200% of the RNI. We compared intake of Vitamin A for children who took a specific supplement known to be higher in vitamin A (Dalivit™, Boston Healthcare Limited) versus any other form of Vitamin A supplementation. Children who received this supplement with vitamin A had significantly higher percentage RNI intakes of Vitamin A (505% RNI vs. 218% RNI, p = 0.014). Similarly, children who received Dalivit™ had higher RNI intake of vitamin D (205% RNI vs. 78% RNI, p = 0.036).

Four children received high doses of iron; however 2 of these had diagnosed iron deficiency anaemia, so were expected to receive therapeutic dosages.

## Discussion

This study found that almost 30% of children from the whole cohort of children with non-IgE mediated food allergies received vitamin and/or mineral supplementation. The mean age of our supplemented cohort was over 5 years with more children receiving a vitamin and/or mineral supplementation when they were on alternative over-the-counter milks (i.e. coconut, oat or rice milk) which are nutritionally not complete [24]. The higher age of the supplemented group is to be expected as a significant number of children in our cohort were below 2 years and on a HF that contain significant amounts of vitamins and minerals [22].

The use of complementary and alternative medicine, including vitamin and mineral supplementation in atopic dermatitis is known to be commonplace in this population [25]. Studies by Johnston et al. [25,26] investigating the use of parent directed dietary elimination for atopic dermatitis, found that 40% of children were on vitamin and/or mineral supplements and that this is more common than in non-atopic children (24%). The key difference between our study and those published on atopic dermatitis were that all of our patients had an individualised dietetic review, whereas in the study by Johnston et al. [24], only 51% had consulted a dietitian. The primary role of the allergy dietitian is not only to discuss elimination of food allergens, but most importantly to suggest food alternatives that replace the nutrients eliminated which would include a HF and other nutritious foods [27].

Not surprisingly, 75% of children on supplements avoided ≥ 2 food allergens, with cow’s milk and soya and a combination of cow’s milk, soya, egg, wheat and others being the most common foods eliminated. It is known that children with multiple food allergies are at a higher risk of poor growth and a deficient vitamin and mineral intake [4,28]. What is of concern is that from our study many children who were identified by a 3-day food diary as having a low intake for certain micronutrients, did not receive a supplement, whereas others received excessive amounts of micronutrients. For example 11 patients from our whole cohort had a deficient vitamin A intake, but only 1 of these received a supplement containing vitamin A; conversely, 21 children with sufficient vitamin A intake, received a supplement containing vitamin A. This has resulted in 59% of our cohort receiving > 200% of the RNI for vitamin A. This occurrence can easily be explained by the UK government prescribable multivitamins which always contain vitamin A. As vitamin D is a commonly reported deficiency in the allergic cohort, all patients that require vitamin D, will therefore automatically receive vitamin A as well [5]. The question is whether the excessive vitamin A intake through supplementation is harmful for the allergic child. In 2003 “tolerable upper limits” for 1–3 year olds of various vitamins and minerals were published by the Scientific Committee on Food in Brussels. In this report the upper limit for vitamin A for this age was set at 800 ug RE/day, which would be 200% of the RNI for this age [29]. Similarly the Upper Safe Limits set by the Institute of Medicine is set at 600 ug/ day for 1–3 year olds and 900 ug/day for 4–9 year olds [30]. However, no observed adverse effects have been reported for intakes up to 6000 ug/day and it is thought that only a chronic dose of 10–20 times the normal dose would lead to toxicity [30]. Levels therefore reported in our study would certainly not lead to toxicity. Excessive intakes of water soluble vitamins like vitamin C, B vitamins and folate are common and rarely are of significant concern, unless the child has reduced fluid intake, abnormal metabolism or metabolic defects [15].

From the whole cohort (supplemented and un-supplemented), 60% of children had a low vitamin D intake, which is not a surprising finding as 52% of those were not on a HF and it is thought that on average only 10% of the daily requirements for vitamin D are contributed by food in older children who are not on breast milk/formula [31,32]. What is of concern in this study is that only 27% of our population with a low dietary intake for vitamin D were identified as at risk and provided with a supplement that contained vitamin D. A recent study by Goldacre et al. [33] indicated that rickets in England is now at its highest in five decades and is not limited to specific ethnic groups, a concern that is reflected in many European countries. Current recommendations in the UK suggest the use of a multivitamin with vitamin D if volume of formula is below 500 ml or if a child is breastfed [34,35]. However, there are no recommendations for vitamin D specifically for children with food allergies, in spite of this being a commonly reported deficiency in this population [36,37].

In this study we also identified children with low intakes of copper, zinc and selenium, which are not commonly associated with IgE-mediated allergies. Deficiencies and low intakes of these minerals have been documented in children with atopic dermatitis and non-IgE mediated gastrointestinal allergies [7,9]. Only 19% of our cohort received supplements with zinc and selenium and none with copper. Meyer et al. [38] has highlighted low intakes of these trace minerals, in particular in children who are not on a hypoallergenic formula. These micronutrients have an important immunomodulatory role and need to be taken into account when assessing dietary intake also in allergic children [39].

Although a low dietary intake may assist in identifying children at risk of a deficiency, it is known that certain nutritional blood markers may be more sensitive and specific in identifying true deficiency. From our cohort only 7 children had biochemical markers of micronutrient intake available to guide dietitians in their supplement recommendation. The reality therefore is that the dietitian will have to rely on dietary intake and foods eliminated from the allergic child’s diet to guide a vitamin and/or mineral supplementation regime. The validity of dietary intake assessment through food records, 24 hour recalls and food frequency questionnaires has been debated by many studies and have significant limitations on an individual level [16]. A systematic review in 2009 on dietary assessment methods for micronutrient intake in infants and children found that a weighed dietary record was more accurate than a food frequency questionnaire for vitamin and mineral intake [16]. In clinical practice however, these are rarely available to a dietitian at the time of the appointment, are time consuming to complete for parents, and for dietitians to analyse [17]. As clinical time constraints are increasing and the child’s nutritional safety is paramount, this study has raised the question for the first time, whether dietitians and clinicians dealing with paediatric food allergies should consider routine vitamin and/or mineral supplements in the light of deficient intake being so common, especially for vitamin D, calcium, zinc and selenium and toxicities (if supplemented within the RNI/RDA) being theoretical rather than of clinical significance.

The limitations of this study are linked to the dietary assessment methods, the analysis program as well as the low number of children on supplements. A 3-day estimated food diary may not be the most accurate dietary assessment method to reflect usual intake. However, in the absence of a validated food frequency questionnaire for allergic children and also the ability to take repeated 24 hour recalls, which would have been affected by the atopic disease process itself, a 3-day food dairy was the best choice. In addition, a 3-day food diary suggest low intake, but certainly would not indicate a vitamin or mineral deficiency. Future studies should aim to assess the association between dietary intake methods and actual deficiencies in the food allergic population. In addition, it may be that parents did not document all the supplements their child was on, due to a concern that the dietitian would request them to discontinue or change the supplements. Due to no RNI being available for vitamin D > 4 years of age we also had to use a cut-off of 10 ug/day for children older than 5 years. This reference value may have elevated the number of children with deficient intake, as it is generally used as an aim value for adults. In addition whilst the dietary analysis program calculates vitamin D intake from the diet, it cannot account for the contribution of sunlight (Zipitis et al. [32]). In this study only 32 children out of 110 that had vitamin/and or mineral supplementation. Although this can be perceived as a low number, we do believe that in spite of this, the study is important in highlighting the difficulties dietitians have in predicting which child requires supplementation of vitamins, minerals or both, and opens the debate to routine supplementation of allergic children irrespective of their food allergy.

## Conclusion

This study investigates the current practice in a tertiary gastroenterology centre of vitamin and/or mineral supplementation in children with non-IgE mediated allergies on an elimination diet. It is the first study that highlights the difficulties in making a decision in regard to appropriate dietary supplementation based on the information a dietitian has during their clinic appointment. We have highlighted that there is a significant discrepancy between children identified with a low intake through a 3-day food diary and those receiving a micronutrient supplement. In the light of vitamin and mineral deficiencies being common in the food allergic cohort, future studies should be performed to assess the impact of routine vitamin and mineral supplementation in children with food allergies using both nutritional blood markers and dietary intake methods.

## Abbreviations

HF: Hypoallergenic formula
IgE: Immunoglobulin E
NHS: National Health Service
RDA: Recommended dietary allowance
RNI: Recommended nutrient intake
